# Supplementary material for: EJPT abstract book for Special issue: Psychotrauma update
Source: Eur J Psychotraumatol. 2015 Apr 8;6:10.3402/ejpt.v6.27953. doi: 10.3402/ejpt.v6.27953 (PMC4393540; doi:10.3402/ejpt.v6.27953)
Supplement: Related texts

# Sub article: Optimizing the efficacy of exposure in PTSD treatment

**DOI:** 10.3402/ejpt.v6.27628

**Published:** 2015-04-08

**Authors:** Gert-Jan Hendriks, Rianne De Kleine, Agnes Van Minnen

**Affiliations:** 1 NijCare, Behavioural Science Institute, Radboud University Nijmegen, Nijmegen, The Netherlands; 2 Center for Anxiety Disorders Overwaal, Institution for Integrated Mental Health Care Pro Persona, Nijmegen, The Netherlands; 3 Department of Psychiatry, Radboud University Medical Centre, Nijmegen, The Netherlands

Both prolonged exposure (PE) and eye movement desensitization reprocessing (EMDR) are recommended in international guidelines as first choice treatments in PTSD patients. Its efficacy is showed in many randomized controlled trials and several meta-analytic reviews (Powers, Halpern, Ferenschak, Gillihan, & Foa, 2010). The proposed working mechanism in PE is fear extinction by effective emotional processing of traumatic memories. Notwithstanding the efficacy of PE, there is room for improvement. Many patients remain symptomatic after treatment (Schnurr et al., 2007), and dropout-rates are substantial and vary between 20 and 35% (Schnurr et al., 2014). Previous studies showed that adding psychological interventions to PE did not succeed in increasing effect-sizes compared to stand-alone PE (Kehle-Forbes et al., 2012). An alternative way to enhance PE efficacy is the combination with pharmacological treatment such as antidepressants, or oxytocin (see this issue: Olff et al., 2015). However, empirical data regarding the efficacy of combining PE with antidepressants are scarce. Additionally, the reported efficacy of adding antidepressants to PE varied between nil and modest compared to PE as stand-alone treatment (De Kleine, Rothbaum, & Van Minnen, 2013; Marshall et al., 2007; Rothbaum et al., 2006; Simon et al., 2008). On the other hand, compared to PE and EMDR the efficacy of pharmacological treatment as stand-alone in PTSD patients is likewise modest (Ipser, Seedat, & Stein, 2006; Watts et al., 2013). Approximately a decade ago an interesting new direction emerged. Fundamental research showed pharmacological enhancement of the underlying mechanisms in exposure therapy: extinction learning and reconsolidation (Debiec & Ledoux, 2004). Although the interrelation between extinction learning and reconsolidation is not fully understood, it is clear that these mechanisms are underlying the efficacy of exposure therapy (Kindt & Soeter, 2013). The findings in fundamental research were translated to clinical studies in anxiety disorders. As fear extinction is linked to the *N*-methyl-d-aspartate (NMDA) glutamatergic receptor activity in the basolateral amygdala, these studies focused on pharmacological enhancement of exposure therapy by NMDA receptor agonists, for instance d-cycloserine (DCS), patients with specific phobia, social anxiety disorder, panic disorder, and obsessive compulsive disorder (Bontempo, Panza, & Bloch, 2012; Norberg, Krystal, & Tolin, 2008). Studies in posttraumatic stress disorder were lacking. Therefore, we conducted a placebo-controlled randomized trial of DCS-enhancement PE treatment in a heterogeneous PTSD population (*n*=67). Although initially DCS-enhancement showed no difference compared to the placebo-group, we found that DCS enhanced PE treatment in a subgroup of patients with more severe PTSD at baseline and an initial non-response on PE (De Kleine, Hendriks, Kusters, Broekman, & Van Minnen, 2012). Furthermore, our results support the influence of personality traits on outcome. High consciousness and low extraversion predicted a better response in the DCS-enhancement treated patients compared to the placebo-group (De Kleine, Hendriks, Smits, Broekman, & Van Minnen, 2014). However, the results of DCS-enhancement in additional PTSD studies are controversial with conflicting results in outcome (Difede et al., 2014; Litz et al., 2012; Rothbaum et al., 2014). The proposed DCS-enhancement in exposure therapy as a one-size-fits-all enhancement strategy is unequivocally too optimistic.

## References

[CIT0001_27628] Bontempo A, Panza K. E, Bloch M. H (2012). D-cycloserine augmentation of behavioral therapy for the treatment of anxiety disorders: A meta-analysis. The Journal of Clinical Psychiatry.

[CIT0002_27628] Debiec J, Ledoux J. E (2004). Disruption of reconsolidation but not consolidation of auditory fear conditioning by noradrenergic blockade in the amygdala. Neuroscience.

[CIT0003_27628] De Kleine R. A, Hendriks G. J, Kusters W. J, Broekman T. G, Van Minnen A (2012). A randomized placebo-controlled trial of D-cycloserine to enhance exposure therapy for posttraumatic stress disorder. Biological Psychiatry.

[CIT0004_27628] De Kleine R. A, Hendriks G. J, Smits J. A, Broekman T. G, Van Minnen A (2014). Prescriptive variables for d-cycloserine augmentation of exposure therapy for posttraumatic stress disorder. Journal of Psychiatric Research.

[CIT0005_27628] De Kleine R. A, Rothbaum B. O, Van Minnen A (2013). Pharmacological enhancement of exposure-based treatment in PTSD: A qualitative review. European Journal of Psychotraumatology.

[CIT0006_27628] Difede J, Cukor J, Wyka K, Olden M, Hoffman H, Lee F. S (2014). D-cycloserine augmentation of exposure therapy for post-traumatic stress disorder: A pilot randomized clinical trial. Neuropsychopharmacology.

[CIT0007_27628] Ipser J, Seedat S, Stein D. J (2006). Pharmacotherapy for post-traumatic stress disorder—A systematic review and meta-analysis. South African Medical Journal.

[CIT0008_27628] Kehle-Forbes S. M, Polusny M. A, MacDonald R, Murdoch M, Meis L, Wilt T. J (2012). A systematic review of the efficacy of adding non exposure components to exposure therapy for posttraumatic stress disorder. Psychological Trauma: Theory, Research, Practice and Policy.

[CIT0009_27628] Kindt M, Soeter M (2013). Reconsolidation in a human fear conditioning study: A test of extinction as updating mechanism. Biological Psychology.

[CIT0010_27628] Litz B. T, Salters-Pedneault K, Steenkamp M. M, Hermos J. A, Bryant R. A, Otto M. W (2012). A randomized placebo-controlled trial of D-cycloserine and exposure therapy for posttraumatic stress disorder. Journal of Psychiatric Research.

[CIT0011_27628] Marshall R. D, Lewis-Fernandez R, Blanco C, Simpson H. B, Lin S. H, Vermes D (2007). A controlled trial of paroxetine for chronic PTSD, dissociation, and interpersonal problems in mostly minority adults. Depression and Anxiety.

[CIT0012_27628] Norberg M. M, Krystal J. H, Tolin D. F (2008). A meta-analysis of D-cycloserine and the facilitation of fear extinction and exposure therapy. Biological Psychiatry.

[CIT0013_27628] Olff M, Van Zuiden M, Koch S, Nawijn L, Frijling J, Veltman D (2015). Intranasal oxytocin: Miracle cure after trauma?. European Journal of Psychotraumatology.

[CIT0014_27628] Powers M. B, Halpern J. M, Ferenschak M. P, Gillihan S. J, Foa E. B (2010). A meta-analytic review of prolonged exposure for posttraumatic stress disorder. Clinical Psychology Review.

[CIT0015_27628] Rothbaum B. O, Cahill S. P, Foa E. B, Davidson J. R, Compton J, Connor K. M (2006). Augmentation of sertraline with prolonged exposure in the treatment of posttraumatic stress disorder. Journal of Traumatic Stress.

[CIT0016_27628] Rothbaum B. O, Price M, Jovanovic T, Norrholm S. D, Gerardi M, Dunlop B (2014). A randomized, double-blind evaluation of D-cycloserine or alprazolam combined with virtual reality exposure therapy for posttraumatic stress disorder in Iraq and Afghanistan war veterans. The American Journal of Psychiatry.

[CIT0017_27628] Schnurr P. P, Chard K. M, Ruzek J. I, Chow B. K, Shih M. C, Resick P. A (2014). Design of VA Cooperative Study #591: CERV-PTSD, comparative effectiveness research in veterans with PTSD. Contemporary Clinical Trials.

[CIT0018_27628] Schnurr P. P, Friedman M. J, Engel C. C, Foa E. B, Shea M. T, Chow B. K (2007). Cognitive behavioral therapy for posttraumatic stress disorder in women: A randomized controlled trial. JAMA.

[CIT0019_27628] Simon N. M, Connor K. M, Lang A. J, Rauch S, Krulewicz S, LeBeau R. T (2008). Paroxetine CR augmentation for posttraumatic stress disorder refractory to prolonged exposure therapy. The Journal of Clinical Psychiatry.

[CIT0020_27628] Watts B. V, Schnurr P. P, Mayo L, Young-Xu Y, Weeks W. B, Friedman M. J (2013). Meta-analysis of the efficacy of treatments for posttraumatic stress disorder. The Journal of Clinical Psychiatry.

# Sub article: The place of trauma in the treatment of personality disorders

**DOI:** 10.3402/ejpt.v6.27629

**Published:** 2015-04-08

**Authors:** Theo J. M. Ingenhoven

**Affiliations:** Center for Psychotherapy, Pro Persona, Lunteren, The Netherlands

In DSM-5, the classification of posttraumatic stress disorder (PTSD) has broadened its scope. The stressor criterion is more explicit with regard to events that can be qualified as a traumatic experience. Moreover, the specifier “with dissociative symptoms” was introduced for individuals experiencing depersonalization or derealization. However, this extension does not fully cover the more profound symptomatic expressions and developmental trajectories of severe and lasting (childhood) abuse and neglect, nor their impact on the development of self and interpersonal functioning as described for personality disorders (as in the alternative DSM-5 model in Section III). During the last decades a number of concepts have been suggested to describe these more profound phenomena: type II disorder (Terr, 1991), complex PTSD (Herman, 1992), enduring personality change after catastrophic experience (WHO, 1992), PTSD related to childhood abuse (Cloitre, Koenen, Cohen, & Han, 2002), complex posttraumatic self-dysregulation (Ford, Courtois, van der Hart, & Nijenhuis, 2005), disorders of extreme stress, not otherwise specified (DESNOS; van der Kolk, Roth, Pelcovitz,Sunday, & Spinazzola, 2005), and developmental trauma disorder (van der Kolk, 2005). However, from a developmental perspective, the etiological role of trauma in complex PTSD is anything but simple. Pre-trauma factors are significant (genetic vulnerability) and what happens after the trauma has the biggest impact (context)! Unfortunately, none of these concepts were incorporated within the new version of the official APA classification system.

With respect to the treatment of PTSD, cognitive behavioral therapies currently dominate the field of treatment approaches. Imaginary exposure and Eye Movement Desensitization and Reprocessing (EMDR) are straightforward evidence-based interventions, widely available to diminish symptoms like intrusive distressing memories, hyper arousal, flash backs, dreams, and nightmares. The aim is to reduce these symptoms within a short period of time.

The majority of PTSD patients (Type I) are characterized by failed prefrontal inhibition of limbic activity. In contrast, however, in the dissociative subtype (feeling zoned out; detached from body) PTSD high prefrontal activation in conjunction with inhibited limbic activation was found.

In complex PTSD, clinicians and patients struggle with long-standing and multifaceted problems (especially suffering from personality disorders, dissociative disorders, mood disorders, and somatic symptom-related disorders), more eclectic approaches are needed integrating emotion regulation and interpersonal functioning strategies as well as psychodynamic understanding. Especially when childhood sexual or physical abuse was long lasting, repetitive, and induced by attachment figures, and when emotional support was lacking, the impact on personality development can be so devastating that other treatment efforts are necessary to establish a trustful therapeutic relation as a starting point for cautious exploration. Evidence-based psychotherapeutic models for (borderline) personality disorders all describe efforts to treat PTSD symptoms within such a specific psychotherapeutic frame of reference. Most of these treatments are intensive (minimal once or twice a week) and long lasting (years). In dialectical behavior therapy (DBT), the initial phase of treatment explicitly focuses on stabilizing by diminishing para-suicidal and self-destructive behaviors, as well as treatment-interfering behaviors. Individual cognitive treatment is combined with skill training to improve emotion regulation. As soon as the patient is skilled and stabilized (most of the time after an initial first year of treatment), DBT will next focus on the treatment of PTSD, using cognitive behavioral approaches like imaginary exposure. During transference focused psychotherapy (TFP), in the mid-phase of treatment, the theme of abuse will be activated within the transference (split self and object representations centered around aggression and hatred). Identification with both victim and perpetrator is elaborated, and sexual and aggressive impulses should be disentangled, with the purpose of resolving inner conflicts, fostering identity integration, and enhancing adaptive functioning. Also in schema-focused therapy (SFT), the treatment of trauma and PTSD is not part of the initial phase of treatment. The first phase of treatment involves identifying maladaptive schemas and building up adaptive capacities (healthy adult mode). Enough social support is a prerequisite to focus on specific former traumatic experiences. SFT describes specific strategies for treating traumatic experiences, like experiential techniques and imagination with rescripting. In mentalization-based treatment (MBT), the concept of mentalizing is used to broaden the perspective on trauma treatment. Mentalizing goes offline when defensive (fight–flight–freeze) responses become activated, promoting rapid responses to imminent danger. In particular, the impact of “attachment trauma” on emotion regulation and mentalizing reflects a dual liability (extreme distress plus impaired development emotion regulation capacities). Later in life, trauma-triggered hyperactivation of the attachment system, and the failure to mentalize, induces primitive modes of thought: psychic equivalence mode, pretend mode, and teleological mode. According to the MBT treatment, model treatment involves far more than processing traumatic memories. Treatment helps the patient to help the self to mentalize trauma and relationship conflicts, in order to develop more secure attachments. The patient uses the therapist as a mirror to understand the self: a “surrogate prefrontal cortex (PFC)”; it provides a buffer between feeling and action: a “pause button”, an opportunity for the patient to reconstruct his or her narrative within a safe and containing environment (first priority), and grounded in reality. In this way, the therapist often becomes the “object of hope.”

What these evidence-based treatments for (borderline) personality disorder have in common is their clear contract setting, their supportive common factors, their efforts to first stabilize their patients before exposing them to traumatic memories, their focus on maintaining a trustworthy therapeutic relationship, and their efforts to tailor psychotherapeutic strategies toward specific vulnerabilities and capabilities to regulate emotions and control (self-destructive) impulses. So, in complex PTSS and personality disorders, the central therapeutic task is NOT specifically to work with the content of traumatic events, but rather involves supporting a mentalizing stance in relation to the meaning and effect of trauma. The focus is primarily on the patients’ mind, not on the event, on the process rather than the content. Over and beyond the holding environment of the therapeutic relation, phase specific and carefully tailored, symptom-focused approaches like exposure and EMDR can reduce typical PTSD symptoms.

## References

[CIT0001_27629] Terr L. C (1991). Childhood traumas: an outline and overview. American Journal of psychiatry.

[CIT0002_27629] Herman J. L (1992). Complex PTSD: a syndrome in survivors of prolonged and repeated trauma. J Trauma Stress.

[CIT0003_27629] WHO (1992). International Statistical Classification of diseases and Related Health problems.

[CIT0004_27629] Cloitre M, Koenen K. C, Cohen L. R, Han H (2002). Skills training in affective and interpersonal regulation followed by exposure: A phase-based treatment for PTSD related to childhood abuse. Journal of Consulting and Child Psychology.

[CIT0005_27629] Ford J. D, Courtois C. A, van der Hart O, Nijenhuis E. R. S (2005). Treatment of complex posttraumatic self-dysregulation. Journal of Traumatic Stress.

[CIT0006_27629] van der Kolk B. A (2005). Developmental trauma disorder. towards a rational diagnosis for children with complex trauma histories. Psychiatric Annals.

[CIT0007_27629] van der Kolk B. A, Roth S, Pelcovitz D, Sunday S, Spinazzola J (2005). Disorders of extreme stress: The empirical foundation of a complex adaptation to trauma. Journal of Traumatic Stress.

# Sub article: Trauma treatment for children and adolescents: stabilizing or trauma-focused therapy?

**DOI:** 10.3402/ejpt.v6.27630

**Published:** 2015-04-08

**Authors:** Ramón J. L. Lindauer

**Affiliations:** 1 Department of Child and Adolescent Psychiatry, Academic Medical Centre, University of Amsterdam, Amsterdam, The Netherlands; 2 De Bascule, Academic Centre for Child and Adolescent Psychiatry, Amsterdam, The Netherlands

Child abuse is widely prevalent in the Netherlands, and the strong social and political focus now put on the issue is fully warranted. Prevalence studies have estimated that around 3% of all Dutch children experience some form of abuse or neglect (Alink et al., 2011; Lamers-Winkelman, Slot, Bijl, & Vijlbrief, 2007; Van IJzendoorn et al., 2007). The most common forms of maltreatment are neglect and domestic violence. Being a witness to domestic violence is a form of child maltreatment. In 2012, approximately 6,000 children under age 18 were accommodated in some kind of women's or homeless facility in the Netherlands. Half of these were in women's refuges after fleeing domestic violence. Research by Brilleslijper-Kater et al. (2010) found that such children had experienced an average of seven potentially distressing or traumatic events; most had directly experienced domestic violence by witnessing verbal or physical violence between their parents. A study in the Therapeutic Foster Care treatment program at De Bascule found that nearly a quarter of such children had experienced five or more different placements outside the parental home. Persistent relocations like these have an adverse impact on child development.

Maltreatment leads to serious problems in children's psychosocial functioning. Mental health problems may include posttraumatic stress disorder, other anxiety disorders, depressive disorder, attachment disorder, and conduct disorder. Such conditions are detrimental to a child's development and may culminate in problems in the family, problems at school, and problems with other children (Jonkman, Verlinden, Bolle, Boer, & Lindauer, 2013; Lindauer & Boer, 2012). The negative consequences may even persist into adulthood (Edwards, Holden, Anda, & Felitti, 2003); adults who have experienced abuse as children have higher risks of physical and psychological problems. Many mothers that experience intimate partner violence develop mental health problems like posttraumatic stress disorder, depressive disorder, or emotional regulation problems. Those problems, in turn, may affect their parenting competence and their children's psychosocial development. Such mothers are also more at risk of maltreating their own children (Dubowitz & Bennett, 2007). In sum, then, the prevalence of child abuse is high, and the short- and long-term consequences are severe.

The first objective of treatment is to create safety within the family. An end must be put to the traumatizing situation. That requires a multidisciplinary individual and systemic strategy that focuses both on the victim or victims and the perpetrator.

In forms of maltreatment such as violence or sexual abuse within the family, treatment must focus both on ensuring safety in the family and on assessing the childrearing capabilities of the parent or parents. Some questions that need to be asked are: (1) Do the parents acknowledge that their child has problems? (2) Do they acknowledge the part that they themselves have played in the onset of the problems? (3) Are they motivated in any way to try to change the situation? (4) Are the parents capable of recognizing experts as people that can truly help? (5) Do the parents themselves have mental health problems that complicate the treatment? Some situations are simply too unsafe, and some parents are utterly incapable of bringing up their child or children. That necessitates the placement of children away from home.

Once a safe environment has been created, the next question is whether the traumatized child should receive trauma-focused treatment right away or whether the first priority should be to ensure a degree of stability. There is considerable debate about this in the adult literature (e.g., De Jongh & ten Broeke, 2014; Dorrepaal et al., 2014). Several stabilization interventions for traumatized children and adolescents are available in the Netherlands, including the insight-therapeutic Stapstenen (“stepping stones”), the drama-therapeutic Tijd voor Toontje, the dissociation-focused Slapende Honden? Wakker Maken! (“don't let sleeping dogs lie”), and the Attachment, Self-Regulation and Competency (ARC) model, but their effectiveness has not been demonstrated. Some empirically validated trauma treatment modalities are trauma-focused cognitive-behavioral therapy (TF-CBT) and eye movement desensitization and reprocessing (EMDR; Diehle, Opmeer, Mannarino, Boer, & Lindauer, 2015); TF-CBT has been more thoroughly evaluated in randomized controlled studies. If a traumatized child or adolescent is broadly capable of talking about what he or she has experienced, then it is preferable to begin an evidence-based trauma intervention immediately without prior stabilization.

## References

[CIT0001_27630] Alink L, Van IJzendoorn R, Bakermans-Kranenburg M, Pannebakker F, Vogels T, Euser S (2011). De tweede nationale prevalentiestudie mishandeling van kinderen en jeugdigen *[Child maltreatment in the Netherlands in 2010: The second Netherlands prevalence study on maltreatment of children and youth, NPM–2010]*.

[CIT0002_27630] Brilleslijper-Kater S, Beijersbergen M, Asmoredjo C, Jansen C, Wolf J (2010). Meer dan bed, bad, broodje pindakaas. Profiel, gezondheid, welzijn en begeleiding van kinderen in de vrouwenopvang en maatschappelijke opvang *[Not just a bed, bath and peanut butter sandwich: Characteristics, health, well-being and care for children growing up with their parent(s) in shelters for homeless people or female victims of domestic violence]*.

[CIT0003_27630] De Jongh A, Ten Broeke E (2014). Response to “treatment compliance and effectiveness in complex PTSD patients with co-morbid personality disorder undergoing stabilizing cognitive behavioral group treatment: a preliminary study.”. European Journal of Psychotraumatology.

[CIT0004_27630] Diehle J, Opmeer B. C, Mannarino A, Boer F, Lindauer R. J. L (2015). Trauma-focused cognitive behavioral therapy or eye movement desensitization and reprocessing: What works in children with posttraumatic stress symptoms? A randomized controlled trial. European Journal of Child and Adolescent Psychiatry.

[CIT0005_27630] Dorrepaal E, Thomaes K, Smit J, Veltman D, Hoogendoorn A, Van Balkom A (2014). Response to “treatment compliance and effectiveness in complex PTSD patients with co-morbid personality disorder undergoing stabilizing cognitive behavioral group treatment: A preliminary study”—Authors’ reply. European Journal of Psychotraumatology.

[CIT0006_27630] Dubowitz H, Bennett S (2007). Physical abuse and neglect of children. Lancet.

[CIT0007_27630] Edwards V. J, Holden G. W, Anda R. F, Felitti V. J (2003). Relationship between multiple forms of childhood maltreatment and adult mental health in community respondents: Results from the Adverse Childhood Experiences (ACE) study. American Journal of Psychiatry.

[CIT0008_27630] Jonkman C. S, Verlinden E, Bolle E. A, Boer F, Lindauer R. J. L (2013). Traumatic stress symptomatology after child maltreatment and single traumatic events: Different profiles. Journal of Traumatic Stress.

[CIT0009_27630] Lamers-Winkelman F, Slot N. W, Bijl B, Vijlbrief A. C (2007). Scholieren over mishandeling. Resultaten van een landelijk onderzoek naar de omvang van kindermishandeling onder leerlingen van het voortgezet onderwijs *[Pupils and maltreatment: Results of a national study on the degree of child maltreatment among pupils of secondary schools]*.

[CIT0010_27630] Lindauer R. J. L, Boer F (2012). Trauma bij kinderen *[Trauma in children]*.

[CIT0011_27630] Van IJzendoorn M. H, Prinzie P, Euser E. M, Groeneveld M. G, Brilleslijper-Kater S. N, Van Noort-Van der Linden A. M. T (2007). Kindermishandeling in Nederland anno 2005: De nationale prevalentiestudie mishandeling van kinderen en jeugdigen (NPM–2005) *[Prevalence of child maltreatment in the Netherlands, 2005: The national prevalence study on maltreatment of children and youth, NPM–2005]*.

# Sub article: Intranasal oxytocin: miracle cure after trauma?

**DOI:** 10.3402/ejpt.v6.27631

**Published:** 2015-04-08

**Authors:** Miranda Olff, Mirjam Van Zuiden, Saskia B. J. Koch, Laura Nawijn, Jessie L. Frijling, Dick J. Veltman

**Affiliations:** 1 Department of Psychiatry, Academic Medical Center, University of Amsterdam, Amsterdam, The Netherlands; 2 Arq Psychotrauma Expert Group, Diemen, The Netherlands; 3 Department of Psychiatry, VU University Medical Center, Amsterdam, The Netherlands

***Background***: In popular media and on Internet, the neuropeptide oxytocin is often advertised as a miracle drug that cures all types of disorders, reduces stress, saves marriages, all conveniently with a nasal spray. Here we will present the effects of intranasal oxytocin on brain function in recently traumatized individuals and patients with posttraumatic stress disorder (PTSD) and discuss clinical implications and further research. PTSD is characterized by exaggerated fear responses to threat and trauma-related stimuli, reflected in altered neural salience processing and emotion regulation (Koch et al., 2014). In addition, many PTSD patients report anhedonia, emotional numbing, and social detachment, reflected in decreased reward sensitivity (Nawijn et al., 2015). Interestingly, dysregulations in these domains also appear to be associated with increased PTSD risk upon trauma exposure and treatment non-response. Currently, there still is a high need for development of effective preventive interventions for PTSD, that can be administered early after trauma, as well as a need for novel adjuvant interventions that augment treatment response to evidence-based psychotherapy for PTSD (i.e., medication-enhanced psychotherapy [MEP]) (see also Hendriks & De Kleine, 2015).

In 2011, we received a large grant (ZonMW TOP) to perform a series of functional neuroimaging studies on the effects of a single intranasal administration of the neuropeptide oxytocin on neural emotional and reward processing in PTSD patients versus healthy traumatized controls, and in recently traumatized individuals at high risk for PTSD due to high levels of distress acutely after trauma.

These studies were based on literature showing that intranasal oxytocin impacted a variety of the behavioral, neural, and neuroendocrine dysregulations observed in PTSD patients and individuals vulnerable for PTSD. Thus, intranasal oxytocin appeared to be a promising candidate for PTSD prevention and augmentation of treatment response (Olff, 2012; Olff, Langeland, Witteveen, & Denys, 2010).

***Objective***: We aimed to investigate the neurobiological mechanisms of fear (i.e., salience processing and emotion regulation) and reward processing underlying oxytocin's potential therapeutic effect for PTSD prevention and treatment. Regarding neural salience processing and emotion regulation, it had been found that oxytocin administration in healthy individuals dampened amygdala reactivity toward threat-related stimuli (Kirsch et al., 2005) and toward conditioned stimuli associated with receiving shocks (Petrovic, Kalisch, Singer, & Dolan, 2008). Therefore, we hypothesized that oxytocin administration would dampen exaggerated neural fear of salience processing and would increase connectivity within neural emotion regulation networks in both PTSD patients and in recently traumatized individuals at increased PTSD risk (Frijling et al., 2012; Frijling et al., 2014). Regarding neural reward processing, it appeared that oxytocin administration increased sensitivity for positive social stimuli by stimulating key brain areas of the reward pathway (such as the nucleus accumbens), a system of brain areas important for processing of positive stimuli, in healthy individuals. Therefore, we hypothesized that oxytocin administration would increase neural sensitivity for (social) reward and thereby affect emotional numbing and social detachment in PTSD patients, allowing PTSD patients to benefit more from provided social support, including the support offered by therapists during treatment (Olff et al., 2010).

***Results***: Here we present a summary of the first results of our fMRI studies on the effects of a single oxytocin administration on fear and reward processing in PTSD patients versus healthy traumatized controls and in recently traumatized individuals.

Our results show that oxytocin administration dampened amygdala reactivity toward emotional faces in PTSD patients, but increased amygdala reactivity toward (negative) emotional faces in both recently traumatized individuals with high levels of distress as well as highly traumatized individuals without psychopathology. Additionally, we found opposing effects of oxytocin administration on functional connectivity of the amygdala with brain areas involved in emotion regulation and salience processing between our study populations. In rest, male PTSD patients showed increased connectivity within the emotion regulation network after oxytocin, while female PTSD patients showed decreased connectivity within the salience network. In contrast, oxytocin administration resulted in decreased resting state connectivity within the emotion regulation network and increased connectivity within the salience network in recently traumatized individuals after exposure to trauma-related script-driven imagery. Regarding neural effects on reward sensitivity, oxytocin administration increased striatal responses to both reward and loss anticipation in PTSD patients and highly traumatized controls.

***Conclusions***: Combined, our findings on the effects of oxytocin administration on neural salience processing and emotion regulation indicate that a single oxytocin administration has differential effects in (recently) traumatized individuals and PTSD patients. This is in line with more recent literature on differential effects of oxytocin administration depending on interpersonal differences and (the interpretation of) the salience of contextual cues (Bartz, Zaki, Bolger, & Ochsner, 2011; Olff et al., 2013). The observed effects of oxytocin administration in our recently traumatized population are contrary to our initial hypothesis, suggesting that caution is warranted regarding administration of oxytocin in recently traumatized individuals. However, for various reasons, it may be expected that repeated oxytocin administration has different effects than a single administration (for review, see Macdonald & Feifel, 2013). Our randomized controlled trial on the effects of a 1-week oxytocin treatment regimen on PTSD prevention, which is currently well underway, will shed more light on the long-term clinical effects of oxytocin administration in recently traumatized individuals.

Our findings on the neural effects of a single oxytocin administration in PTSD patients are in line with our hypotheses. Thus, oxytocin is not a miracle panacea, but our results do support the notion that intranasal oxytocin administration is a promising candidate for augmentation of PTSD treatment response. As a next step, the potential of intranasal oxytocin administration as an adjunctive agent during psychotherapy for PTSD, such as before exposure-based therapies, should be further investigated in a clinical setting, preferably in a randomized placebo-controlled trial.

## Grants and disclosures

The study is supported by grants from ZonMw (Top Grant), the Netherlands organization for Health Research and Development (grant no. 40-00812-98-10041) and the Academic Medical Center Research Council (110614).

All authors declare that they have no biomedical financial interests and no potential conflicts of interest.

## References

[CIT0001_27631] Bartz J. A, Zaki J, Bolger N, Ochsner K. N (2011). Social effects of oxytocin in humans: Context and person matter. Trends in Cognitive Sciences.

[CIT0002_27631] Frijling J, Van Zuiden M, Koch S, Nawijn L, Veltman D, Olff M (2012). Boosting the oxytocin system in acute trauma victims at risk for PTSD: The rationale and design of a randomized controlled trial. European Journal of Psychotraumatology.

[CIT0003_27631] Frijling J. L, Van Zuiden M, Koch S. B. J, Nawijn L, Goslings J. C, Luitse J. S (2014). Efficacy of oxytocin administration early after psychotrauma in preventing the development of PTSD: Study protocol of a randomized controlled trial. BMC Psychiatry.

[CIT0004_27631] Hendriks G. J, De Kleine R (2015). Optimizing the efficacy of exposure in PTSD-treatment. European Journal of Psychotraumatology.

[CIT0005_27631] Kirsch P, Esslinger C, Chen Q, Mier D, Lis S, Siddhanti S (2005). Oxytocin modulates neural circuitry for social cognition and fear in humans. The Journal of Neuroscience.

[CIT0006_27631] Koch S. B. J, Van Zuiden M, Nawijn L, Frijling J. L, Veltman D. J, Olff M (2014). Intranasal oxytocin as strategy for medication-enhanced psychotherapy of PTSD: Salience processing and fear inhibition processes. Psychoneuroendocrinology.

[CIT0007_27631] Macdonald K, Feifel D (2013). Helping oxytocin deliver: Considerations in the development of oxytocin-based therapeutics for brain disorders. Frontiers in Neuroscience.

[CIT0008_27631] Nawijn L, Van Zuiden M, Frijling J. L, Koch S. B, Veltman D. J, Olff M (2015). Reward functioning in PTSD: A systematic review exploring the mechanisms underlying anhedonia. Neuroscience & Biobehavioral Reviews.

[CIT0009_27631] Olff M (2012). Bonding after trauma: On the role of social support and the oxytocin system in traumatic stress. European Journal of Psychotraumatology.

[CIT0010_27631] Olff M, Frijling J. L, Kubzansky L. D, Bradley B, Ellenbogen M. A, Cardoso C (2013). The role of oxytocin in social bonding, stress regulation and mental health: An update on the moderating effects of context and interindividual differences. Psychoneuroendocrinology.

[CIT0011_27631] Olff M, Langeland W, Witteveen A, Denys D (2010). A psychobiological rationale for oxytocin in the treatment of posttraumatic stress disorder. CNS Spectrums.

[CIT0012_27631] Petrovic P, Kalisch R, Singer T, Dolan R. J (2008). Oxytocin attenuates affective evaluations of conditioned faces and amygdala activity. The Journal of Neuroscience.

# Sub article: Substance use disorders, trauma, and PTSD

**DOI:** 10.3402/ejpt.v6.27632

**Published:** 2015-04-08

**Authors:** Wim Van den Brink

**Affiliations:** Department of Psychiatrie, Academic Medical Center, University of Amsterdam, Amsterdam, The Netherlands

Substance use disorders (SUDs) and traumas frequently co-occur. A recent German study found that 66% of the women with a diagnosis of opioid dependence also reported sexual abuse, whereas the figure for men was 11% (Schäfer et al., 2014). In a similar study among alcohol-dependent patients, 35% of the female patients and 6% of male patients were sexually abused as a child (Schäfer et al., 2009). In one of our own studies among treatment-seeking alcohol-dependent patients (80% male), we found the following figures: 24% sexually abused as a child, 15% physically abused as a child, 16% witnessed domestic violence as a child, 42% physically abused as an adult, and 11% sexually abused as an adult (Langeland, Draijer, & Van den Brink, 2002). Therefore, it is hardly surprising that nearly 20% of these patients were diagnosed with a DSM-III-R posttraumatic stress disorder (PTSD). This lecture will focus on the latter group of patients: SUD patients with PTSD.

In the general population, approximately 10% of women and 5% of men meet criteria for a lifetime diagnosis of PTSD. However, for patients with alcohol dependence, the figures are approximately 25% (OR 3.6) and 10% (OR 3.2), respectively. Conversely, about 35% of people diagnosed with PTSD also have an alcohol use disorder (Kessler, Sonnega, Bromet, Hughes, & Nelson, 1995).

Among treatment-seeking SUD patients, 20–50% have a lifetime diagnosis of PTSD, whereas 15–40% met criteria for PTSD in the last year (Brady, Killeen, Brewerton, & Lucerini, 2000; Van Dam, Ehring, Vedel, & Emmelkamp, 2010). Conversely, about 20% of the treatment-seeking patients with PTSD also meet criteria for a current SUD. This is important because this comorbidity is associated with more severe PTSD and more severe SUD and with negative treatment outcomes for both PTSD and SUD (Brady et al., 2000). Furthermore, episodes of “re-experiencing” are associated with an increase in substance abuse and an increased risk of relapse, whereas withdrawal and abstinence are associated with an increase of PTSD symptoms (Brown, Stout, & Gannon-Rowley, 1998).

There are multiple theories about the reasons for the high co-occurrence of SUD and PTSD. Some believe that PTSD precedes SUD and that SUD is the consequence of attempts to self-medicate PTSD symptoms. Others believe that SUD occurs first with substance-use-related traumas as an adult resulting in comorbid PTSD. Finally, some consider that both SUD and PTSD are the result of a shared vulnerability due to some genetic predisposition or early childhood trauma. Research shows that PTSD precedes SUD far more frequently than the other way around, that PTSD and SUD are both very often preceded by trauma in early childhood, and that there is some genetic overlap for the two disorders (Kessler et al., 1995; Sartor et al., 2011). On the basis of these etiological considerations, it seems that the treatment of PTSD (as a causal factor in the development of SUD) is more important than the treatment of SUD in patients with both disorders. However, in the course of the disorder, the course of SUD may become rather independent and simultaneous treatment of both disorders would be most preferable. However, this still raises the question of whether treatment should be (predominantly) psychotherapeutic or pharmacological and, furthermore, what kind of psychotherapy or medication.

A large number of studies have looked at the effect of psychotherapeutic treatments of SUD patients with comorbid PTSD. The results of these studies have been summarised in two recent reviews (Najavits & Hien, 2013; Van Dam, Vedel, Ehring, & Emmelkamp, 2012). Both reviews deal with cognitive behavioural therapy aimed at stabilisation without exposure (such as Seeking Safety) as well as cognitive behavioural therapy with exposure. The latter review contains 35 studies and pays a lot of attention to Seeking Safety (22 studies), a treatment developed by Najavits. The review strongly recommends Seeking Safety as the treatment of choice. The review by Van Dam et al. contains 17 studies and is quite critical about the results of both forms of cognitive behavioural therapy. However, in contrast to Najavits and Hien, it cautiously recommends treatments with exposure, arguing that this form of treatment shows the best results with non-addicts and that it is also more appreciated by comorbid patients. Both reviews admit that none of the psychotherapeutic interventions for PTSD had a clear effect on substance use.

There are various different effective medications available for the treatment of nicotine, heroine, alcohol, and cannabis dependence, and there are some promising medications for the treatment of cocaine and other stimulant use disorders (Van den Brink, 2012). However, the number of studies about the pharmacological treatment of PTSD in patients with a comorbid SUD is rather limited, and the methodological quality of many of these studies is low. Nevertheless, a recent review (Norman et al., 2012) considers the following medications: the antidepressant sertraline, the antipsychotic quetiapine, and the anti-epileptic topiramate. Sertraline seems to work best in comorbid patients with a primary diagnosis of PTSD. However, sertraline might be contraindicated in a primary alcohol-dependent patient with secondary PTSD because in these patients sertraline may lead to increased alcohol use (Brady et al., 2005). The advantage of topiramate is that alcohol detoxification can be initiated at the beginning of the treatment because it prevents withdrawal symptoms and complications as well as reducing substance use and PTSD symptoms.

An important problem that needs more attention is that most interventions have no effect on nightmares or other sleeping problems associated with either PTSD or SUD. A recent review (Nappi, Drummond, & Hall, 2012) concludes that the best psychotherapeutic treatment for nightmares in patients with PTSD is a cognitive behavioural intervention with exposure. Also, one or more of the following medications may be effective: topiramate, prazosin, quetiapine, duloxetine, and gabapentin. It should be noted that topiramate and prazosin are probably also effective in the treatment of alcohol dependence (Simpson et al., 2009; Van den Brink, 2012).

Based on this overview of the literature, the author recommends: 1) all treatment-seeking SUD patients should been screened for PTSD; 2) all treatment-seeking PTSD patients should be questioned about their alcohol and drug use; 3) all patients with PTSD and/or SUD should be screened for the presence of early childhood trauma; 4) the presence childhood trauma should always be validated and possibly treated; 5) the relationship between PTSD symptoms and substance use should be assessed and monitored; 6) the patient's preference for abstinence or controlled use should be taken into account because both drug use and withdrawal can worsen the course of PTSD symptoms; 7) all patients with PTSD and SUD should be screened for sleeping disorders; 8) it is preferable to treat PTSD and SUD simultaneously either with one or two different therapists; 9) cognitive behavioural therapy with exposure is preferred (including eye movement desensitisation and reprocessing), possibly in combination with pharmacotherapy for addiction and/or PTSD; 10) there is no role for benzodiazepines or tricyclic antidepressants in the treatment of patients with SUD and PTSD; and 11) there might be a role for topiramate, prazosin, or gabapentin in the treatment of nightmares in patients with PTSD and SUD.

## References

[CIT0001_27632] Brady K. T, Killeen T. K, Brewerton T, Lucerini S (2000). Comorbidity of psychiatric disorders and posttraumatic stress disorder. Journal of Clinical Psychology.

[CIT0002_27632] Brady K. T, Sonne S, Anton R. F, Randall C. L, Back S. E, Simpson K (2005). Sertraline in the treatment of co-occurring alcohol dependence and posttraumatic stress disorder. Alcoholism: Clinical and Experimental Research.

[CIT0003_27632] Brown P. J, Stout R. L, Gannon-Rowley J (1998). Substance use disorder-PTSD comorbidity. Patients’ perceptions of symptom interplay and treatment issues. Journal of Substance Abuse Treatment.

[CIT0004_27632] Kessler R. C, Sonnega A, Bromet E, Hughes M, Nelson C. B (1995). Posttraumatic stress disorder in the National Comorbidity Survey. Archives of General Psychiatry.

[CIT0005_27632] Langeland W, Draijer N, Van den Brink W (2002). Trauma and dissociation in treatment-seeking alcoholics: Towards a resolution of inconsistent findings. Comprehensive Psychiatry.

[CIT0006_27632] Najavits L. M, Hien D (2013). Helping vulnerable populations: A comprehensive review of the treatment outcome literature on substance use disorder and PTSD. Journal of Clinical Psychology.

[CIT0007_27632] Nappi C. M, Drummond S. P, Hall J. M (2012). Treating nightmares and insomnia in posttraumatic stress disorder: A review of current evidence. Neuropharmacology.

[CIT0008_27632] Norman S. B, Myers U. S, Wilkins K. C, Goldsmith A. A, Hristova V, Huang Z (2012). Review of biological mechanisms and pharmacological treatments of comorbid PTSD and substance use disorder. Neuropharmacology.

[CIT0009_27632] Sartor C. E, McCutcheon V. V, Pommer N. E, Nelson E. C, Grant J. D, Duncan A. E (2011). Common genetic and environmental contributions to post-traumatic stress disorder and alcohol dependence in young women. Psychological Medicine.

[CIT0010_27632] Schäfer I, Verthein U, Oechsler H, Deneke C, Riedel-Heller S, Martens M (2009). What are the needs of alcohol dependent patients with a history of sexual violence? A case-register study in a metropolitan region. Drug and Alcohol Dependence.

[CIT0011_27632] Schäfer I, Gromus L, Atabaki A, Pawils S, Verthein U, Reimer J (2014). Are experiences of sexual violence related to special needs in patients with substance use disorders? A study in opioid-dependent patients. Addictive Behaviors.

[CIT0012_27632] Simpson T. L, Saxon A. J, Meredith C. W, Malte C. A, McBride B, Ferguson L. C (2009). A pilot trial of the alpha-1 adrenergic antagonist, prazosin, for alcohol dependence. Alcoholism: Clinical and Experimental Research.

[CIT0013_27632] Van Dam D, Ehring T, Vedel E, Emmelkamp P. M (2010). Validation of the primary care posttraumatic stress disorder screening questionnaire (PC-PTSD) in civilian substance use disorder patients. Journal of Substance Abuse Treatment.

[CIT0014_27632] Van Dam D, Vedel E, Ehring T, Emmelkamp P. M (2012). Psychological treatments for concurrent posttraumatic stress disorder and substance use disorder: A systematic review. Clinical Psychology Review.

[CIT0015_27632] Van den Brink W (2012). Evidence-based pharmacological treatment of substance use disorders and pathological gambling. Current Drug Abuse Reviews.

# Sub article: PTSD as a memory disorder

**DOI:** 10.3402/ejpt.v6.27633

**Published:** 2015-04-08

**Authors:** Hein van Marle

**Affiliations:** Department of Psychiatry, VU-Medical Centre and GGz inGeest, Amsterdam, The Netherlands

***Psychiatry as clinical neuroscience***: Adopting a cognitive neuroscience perspective on psychiatric disorders is crucial in attaining a deeper understanding of disease pathophysiology and symptomatology and developing novel theory-driven treatments. Worldwide, a great number of neuroscientists study basic brain functions like attention, fear, reward, and affect. The clinical disturbance of these brain functions is in part what we call psychiatry. Yet, most clinicians have very little knowledge of this literature, and intervention research in psychiatry is rarely guided by findings from fundamental neuroscience. In this presentation, I make the claim that post traumatic stress disorder (PTSD) can be considered a disorder of basic memory function. Hence, a more mechanistic account of PTSD symptoms, like flashbacks and hypervigilance, can be derived from fundamental insights into, for instance, the relationship between stress and memory and memory (re)consolidation. Furthermore, these findings (should) guide us in the development of novel treatments for PTSD.

***Memory disturbances in PTSD: focus on consolidation***: Although all phases of the memory cycle play their part in PTSD, the consolidation phase seems to be particularly important, and unlike the encoding phase (trauma acquisition), accessible to therapeutic intervention. During memory consolidation, initially fragile memory traces are reorganized and integrated into long-term storage (McGaugh, 2000). This process occurs when awake, but is particularly facilitated during sleep and can take up to years to fully develop (Diekelmann & Born, 2010). According to systems-level consolidation theory (Frankland & Bontempi, 2005), this reorganization of memory traces is realized through a gradual transfer of information from initial hippocampal-cortical links after encoding to predominant cortico-cortical connections, thereby effectively categorizing and conceptualizing the mnemonic episode.

On a neuronal level, this transfer is achieved through a process called replay during which the same hippocampal-cortical firing patterns that represent the stored memory during awake state are being played back to the cortex while asleep (Skaggs & McNaughton, 1996). This strengthens cortico-cortical associations and integrates the memory into existing cortical memory circuits. Currently, experimenters at the forefront of memory research aim to target this replay process in an attempt to boost memory consolidation. Successful attempts consist of presenting auditory or olfactory cues (sounds or smells) that were linked to memory encoding during subsequent slow wave sleep (e.g., Van Dongen et al., 2012).

In case of emotional memory, current theories suggest that memory consolidation additionally serves to preserve and solidify the declarative, factual aspect of an emotional memory trace, while at the same time depotentiating its affective charge (Walker & Van der Helm, 2009). This progressive decoupling of emotion and memory is thought to be facilitated by stress-related hormones and neurotransmitters (like cortisol and norepinephrine) that act at the level of the amygdala, thereby modulating the hippocampal-cortical transfer (Van Marle, Hermans, Qin, Overeem, & Fernandez, 2013).

***Understanding PTSD symptoms***: In case of traumatic memory as in PTSD, the process of memory consolidation seems to fail. The traumatic memory trace stays primarily located in subcortical and primary perceptual areas, leaving it tightly coupled to its autonomic and perceptual markers, and lacking the appropriate integration in autobiographical, cortical memory networks. Exposure to a trauma trigger subsequently results in a solely involuntarily retrieved memory trace (intrusion), that is very hard to verbalize, often fragmented in time, and consisting for the most part of primary sensory information (images, smell, sounds) that is linked to physiological fear symptoms (Brewin, 2011). Due to the lack of autobiographical context, the memory is relived as happening in the present. Thus a failure to properly consolidate and thus emotionally depotentiate potentially traumatic memories may form the neural basis of key PTSD symptoms like unwanted memories, intrusive flashbacks, nightmares, hyperarousal, and dissociation. Reduction of PTSD symptoms is accomplished by successful transfer to pre-existent, cortical memory circuits.

***From consolidation to reconsolidation***: An exciting development in memory research with clear implications for psychiatry has been the discovery of memory reconsolidation (Nader, Schafe, & Le Doux, 2000). In contrast to the idea that memory once formed is resistant to change, reconsolidation theory holds that after the retrieval or reactivation of a memory, it becomes labile and modifiable. This enables it to be reconsolidated in another form (updated) or prevented from being reconsolidated at all, effectively erasing it from memory. As opposed to memory extinction, in which a new (stronger) safety memory is formed in parallel to the original fear memory, this has the advantage of preventing the spontaneous recovery of fear. The initial experiment of Nader and colleagues showed that (toxically) blocking protein synthesis in the amygdala of the rat after reactivation of a conditioned fear memory led to its eradication from memory. Since then, an ongoing series of parallel experiments in rats and humans have found additional, more applicable methods of intervening in the reconsolidation of associative, aversive memory. Extinction learning itself can be used to update the original memory (Monfils, Cowansage, Klann, & LeDoux, 2009; Schiller et al., 2010), whereas propranolol (blocking noradrenergic action in the brain) disrupts the reconsolidation of original fear memories (Kindt, Soeter, & Vervliet, 2009), providing there is a form of prediction error during reactivation (Sevenster, Beckers, & Kindt, 2013). A critical time window of 10 min to up to 6 h after reactivation was found for all these interventions. Recently, Kroes and colleagues extended reconsolidation theory to naturalistic, episodic, emotional memory (Kroes et al., 2014). Implementing the appropriate control conditions, they showed that reactivation of a learned emotional storyline in depressed patients just prior to electroconvulsive therapy (ECT) treatment resulted in change-level memory of the story after 24 h. Hence, ECT seems to successfully prevent the restorage of emotionally laden information.

***Implications for (future) treatment***: Approaching PTSD as a memory disorder, with a specific focus on (re)consolidation opens up exciting, new ways to think about treatment. Current (pharmacological) intervention studies in PTSD however, in my opinion, fail to adhere to or be inspired by findings from (re)consolidation memory research. Several studies investigate the prolonged administration of propranolol or hydrocortisone for multiple days post-trauma (De Quervain, Aerni, Schelling, & Roozendaal, 2009; Pitman et al., 2002). Independent of the reported (mixed) effects, this leaves you uninformed about the mechanism of action because all phases of the memory cycle, including (spontaneous) retrieval, are affected. Alternatively, drug augmentation studies attempt to enhance the effect of exposure-based psychotherapy by administering drugs, mainly the NMDA receptor agonist d-cycloserine (DCS), in parallel to the therapeutic session. The mixed results (e.g. Litz et al., 2012; Rothbaum et al., 2014) are generally attributed to potentially also augmenting non-effective treatment sessions when the drug is administered prior to the session (Hofmann, Otto, Pollack, & Smits, 2015). Although this is theoretically correct, findings from (re)consolidation research emphasize the importance of timing drug augmentation to first post-session sleep (nap or night), thereby taking into account the critical time window of 10 min to 6 h after reactivation of the trauma memory. Alternatively to DCS, disruptive agents such as propranolol could prevent (as opposed to facilitate) restorage of the trauma memory when administered post-session. Invasive methods such as ECT or transcranial magnetic stimulation (TMS), when following reactivation, could potentially disrupt reconsolidation even more effectively, hypothetically erasing the trauma episode from memory.

Although often less accessible for therapeutic intervention, more research efforts should be directed towards intervening directly during post-trauma sleep, for instance, in emergency rooms or combat situations. The opportunity to influence the first-time transfer of potentially traumatic memory traces, either by administering drugs or just generally improving sleep quality, seems especially valuable.

A promising, but experimental, avenue for treatment is the selective targeting of replay to boost consolidation of trauma memory. Sound or smell stimuli that are linked to original trauma acquisition could be presented time-locked to different sleep stages or events. This patient-specific, trauma-tailored reactivation during sleep could by itself open up a new reconsolidation window, in which again facilitative or disruptive interventions can be applied.

So far, none of these interventions are available for clinical practice, but with a growing interest of clinical researchers in cognitive neuroscience, this is likely to happen sooner rather than later. A critical remaining question in PTSD pertains to whether any intervention aims to facilitate or speed up the (re)consolidation process thereby decoupling memory content from its affective charge (for instance, using DCS in first post-trauma sleep); or alternatively disrupt (re)consolidation of the traumatic memory trace thereby preventing its restorage (for instance, using propranolol or more invasive techniques such as ECT).

## References

[CIT0001_27633] Brewin C. R (2011). The nature and significance of memory disturbance in posttraumatic stress disorder. Annual Review of Clinical Psychology.

[CIT0002_27633] De Quervain D. J, Aerni A, Schelling G, Roozendaal B (2009). Glucocorticoids and the regulation of memory in health and disease. Frontiers in Neuroendocrinology.

[CIT0003_27633] Diekelmann S, Born J (2010). The memory function of sleep. Nature Reviews Neuroscience.

[CIT0004_27633] Frankland P. W, Bontempi B (2005). The organization of recent and remote memories. Nature Reviews Neuroscience.

[CIT0005_27633] Hofmann S. G, Otto M. W, Pollack M. H, Smits J. A (2015). D-cycloserine augmentation of cognitive behavioral therapy for anxiety disorders: An update. Current Psychiatry Reports.

[CIT0006_27633] Kindt M, Soeter M, Vervliet B (2009). Beyond extinction: Erasing human fear responses and preventing the return of fear. Nature Neuroscience.

[CIT0007_27633] Kroes M. C, Tendolkar I, Van Wingen G. A, Van Waarde J. A, Strange B. A, Fernandez G (2014). An electroconvulsive therapy procedure impairs reconsolidation of episodic memories in humans. Nature Neuroscience.

[CIT0018_27633] Litz B. T, Salters-Pedneault K, Steenkamp M. M, Hermos J. A, Bryant R. A, Otto M. W (2012). A randomized placebo-controlled trial of D-cycloserine and exposure therapy for posttraumatic stress disorder. Journal of psychiatric research.

[CIT0008_27633] McGaugh J. L (2000). Memory--a century of consolidation. Science.

[CIT0009_27633] Monfils M. H, Cowansage K. K, Klann E, LeDoux J. E (2009). Extinction-reconsolidation boundaries: Key to persistent attenuation of fear memories. Science.

[CIT0010_27633] Nader K, Schafe G. E, Le Doux J. E (2000). Fear memories require protein synthesis in the amygdala for reconsolidation after retrieval. Nature.

[CIT0011_27633] Pitman R. K, Sanders K. M, Zusman R. M, Healy A. R, Cheema F, Lasko N. B (2002). Pilot study of secondary prevention of posttraumatic stress disorder with propranolol. Biological Psychiatry.

[CIT0019_27633] Rothbaum B. O, Price M, Jovanovic T, Norrholm S. D, Gerardi M, Dunlop B (2014). A randomized, double-blind evaluation of D-cycloserine or alprazolam combined with virtual reality exposure therapy for posttraumatic stress disorder in Iraq and Afghanistan War veterans. The American journal of psychiatry.

[CIT0012_27633] Schiller D, Monfils M. H, Raio C. M, Johnson D. C, Ledoux J. E, Phelps E. A (2010). Preventing the return of fear in humans using reconsolidation update mechanisms. Nature.

[CIT0013_27633] Sevenster D, Beckers T, Kindt M (2013). Prediction error governs pharmacologically induced amnesia for learned fear. Science.

[CIT0014_27633] Skaggs W. E, McNaughton B. L (1996). Replay of neuronal firing sequences in rat hippocampus during sleep following spatial experience. Science.

[CIT0015_27633] Van Dongen E. V, Takashima A, Barth M, Zapp J, Schad L. R, Paller K. A (2012). Memory stabilization with targeted reactivation during human slow-wave sleep. Proceedings of the National Academy of Sciences of the United States of America.

[CIT0016_27633] Van Marle H. J, Hermans E. J, Qin S, Overeem S, Fernandez G (2013). The effect of exogenous cortisol during sleep on the behavioral and neural correlates of emotional memory consolidation in humans. Psychoneuroendocrinology.

[CIT0017_27633] Walker M. P, Van der Helm E (2009). Overnight therapy? The role of sleep in emotional brain processing. Psychological Bulletin.

# Sub article: Fear, helplessness, and horror—if it does not stop: reflections on the evolving concept of impact of trauma

**DOI:** 10.3402/ejpt.v6.27634

**Published:** 2015-04-08

**Authors:** Eric Vermetten

**Affiliations:** 1 Department of Psychiatry, Leiden University Medical Centrum, Leiden, The Netherlands; 2 Department of Defense, Military Mental Health Research, Utrecht, The Netherlands; 3 Arq Psychotrauma Expert Group, Diemen, The Netherlands

War psychiatry has served as the backbone of our current understanding of the impact of psychotrauma. The First World War confronted the world with an invalidating phenotype that has since then seen itself represented with different names, of which shell shock is probably best known. Since then wars have provided us a moral imperative to better understand and prevent the impact of combat (see special issue EJPT, 2014; Yehuda, Vermetten, & McFarlane, 2014). Wars, but also events such as armed robbery, rape, accidents, and natural disasters, can confront an individual with danger to life, injury to oneself or another person, or in the worst case death. We categorize so-called “near death” or “serious injury” experiences as traumatic stressors. For some, they may be associated with intense fear, horror, and helplessness and can cause serious lasting dysregulations of daily life. It can cause a dysfunctional repetitive cycle from which it is not easy to “snap out of” (Bremner & Reed, 2014). For some, life is no longer the same, and one's life narrative cannot stop gravitating toward the traumatic events. The fear, helplessness, and horror as well as of symptoms, nightmares, flashbacks, avoidance, hopelessness, guilt, irritability, memory, and concentration difficulties and sleep problems can serve as clinical signifiers. It lasted until 1980 before and cause the an phenotype twas hat included exists since 1980 in the third edition of the Diagnostic Statistical Manualpsychiatric classification as post-traumatic stress disorder (PTSD) (Van der Kolk, 2014; Vermetten, Kleber, & Van der Hart, 2012).

Since then, three decades of research have passed (Vermetten & Lanius, 2012), in which the number of studies on PTSD have exponentially increased. The studies have moved from identification to validation, treatment, and also prediction, and shifted from tertiary to primary prevention and resilience enhancement. But with the increasing number of studies, the number of questions has also increased. Most importantly, what do we know now what we did not know then? Has our knowledge led to a greater understanding by mental health professionals? Has it been incorporated in the educational curricula of universities? Has the knowledge led to new treatment opportunities? Can you “heal” or “cure” PTSD?

A century ago, the term “shell shock,” a medical label, was introduced to represent the ones with severe disturbances due to exposure to shells of the war. Yet, there was no good concept for the disorder, because it occurred also in soldiers that had not been exposed to shells. The Kriegstraumatische Zitterneurose, as Germans labeled it, left room for controversy as this did not uniquely focus on external causes. A world war later, with devastating experiences of the Holocaust and names like “physioneurosis” and “KZ syndrome,” we became even more aware of the unique attribution of the clinical phenomenology to traumatic stress, and moved away from “mental breakdown” or “moral weakness” (Weisaeth, 2002). Yet it took many more years to recognize the full impact of traumatic events as precipitating events and find a discourse within neurosciences with notions of, for example, allostasis (McEwen, 1988) and resilience (Bonanno, 2004). Pivotal was the Post–Vietnam War movement that helped embrace the lasting impact of trauma exposure in DSMIII. Salience did not lead to simplicity. Despite preservation of the name PTSD and moving away from the category of anxiety disorders in DSM5, it has not become easier, or to say the least “simple.”

To list a few challenges:

- The current DSM5 lists 23 symptoms that need to be present in a variety of combinations to qualify someone as suffering from PTSD, contributing to a heterogeneity of the disorder.
- New questions emerge about the complexity of the disorder, for example, the dissociative element, and discussions of “simple” vs “complex”.
- The majority of people exposed to trauma will not be clinically affected. There are important notions about resilience, what drives resilience?
- We revisit questions that drove the post war period of the First World War about stigma, moral weakness, or injury, and the discrimination of PTSD with “visible” disorders such as mild traumatic brain injury (mTBI).
- The PTSD of today is a grimsy disorder, does not have a clear incubation time, and varies in its presentation in relation to the time of onset after exposure.
- It is a disorder that for diagnosis is purely based on self-disclosure, self-observation. We seem to completely go beyond behavioral observations as important assessment elements, and do not use hetero-anamnestic information as a clinical or critical source of information.

Perhaps most important of all, despite promising new findings, there is also still no “biological qualifier,” which serves to determine who is more or less likely to get it, if exposed. No “mental Cooper test” exists, that can help us to select who is susceptible to the disorder. We invest many resources since it is felt that this prediction will not only be possible but also desperately wanted. Militaries and other uniformed services are dying to know how to implement this in selection procedures (Yehuda et al., 2014), saving young men and women from unnecessary suffering. How far are we from first implementation of biomarkers in selection procedures? Do we see breakthroughs emerging to support this? In our fourth decade of research since 1980 with rapid developments in (epi)genetics, optogenetics, and novel imaging methods, this may be not that far down the road (Vermetten, Baker, & Yehuda, 2015; Vermetten, Zohar, & Krugers, 2014).

Psychological trauma has become an iconic element in our society. Starting little over 100 years ago, psychotraumology is like a fast moving train, with currently more than 2,500 scientific publications per year on the topic of PTSD. The field is presenting itself to a highly modernized world, where stakes are high and education of a discourse is highly needed, because trauma is not likely to disappear. The artificial dichotomy between vulnerability and resilience is challenged by increasing violence, natural, and man-made disasters, and by participation in small conflicts and big-scaled wars. Murray and Lopez predicted in the Lancet in 1997 that in 2020 war and violence would be ranked in the top 10 of “disease burden” (Murray & Lopez, 1997). If they are right, this puts a burden on the society. Do we habituate? Or sensitize? Yet in some countries, academia is hesitant to embrace or acknowledge the impact for the small group that is suffering, or one is not thinking yet in terms of healing and supportive communities or societies. Maybe the most important lesson we learned over the last years is to acknowledge trauma, identify the ones affected in need, and prevent the “engraving” of the impact of the stress early. In order to do so, we must be able to identify the persons that are biologically vulnerable representing a vulnerable phenotype, in order to justify early treatment, or monitor the developmental course in the aftermath of trauma exposure recommendation and facilitate his or her empowerment.

## References

[CIT0001_27634] Bonanno G. A (2004). Loss, trauma, and human resilience: Have we underestimated the human capacity to thrive after extremely aversive events?. The American Psychologist.

[CIT0002_27634] Bremner J. D, Reed J. D (2014). You can't just snap out of it. The real path to recovery from psychological trauma.

[CIT0003_27634] McEwen B. S (1998). Stress, adaptation, and disease: Allostasis and allostatic load. Annals of the New York Academy of Sciences.

[CIT0004_27634] Murray C. J, Lopez A. D (1997). Global mortality, disability, and the contribution of risk factors: Global Burden of Disease Study. Lancet.

[CIT0005_27634] Van der Kolk B (2014). The body keeps the score. Brain, mind and body in the healing of trauma.

[CIT0006_27634] Vermetten E, Baker D, Yehuda R (2015). New findings from prospective studies. Psychoneuroendocrinology.

[CIT0007_27634] Vermetten E, Kleber R, Van der Hart O (2012). Handboek Posttraumatisch Stressstoornissen [Handbook Posttraumatic Stress Disorders].

[CIT0008_27634] Vermetten E, Lanius R. A (2012). Biological and clinical framework for posttraumatic stress disorder. Handbook of Clinical Neurology.

[CIT0009_27634] Vermetten E, Zohar J, Krugers H (2014). Pharmacotherapy in the aftermath of trauma; opportunities in the ‘golden hours’. Current Psychiatry Reports.

[CIT0010_27634] Weisaeth L (2002). The European history of psychotraumatology. Journal of Traumatic Stress.

[CIT0011_27634] Yehuda R, Vermetten E, McFarlane A (2014). PTSD in the military: Special considerations for understanding prevalence, pathophysiology and treatment following deployment. European Journal of Psychotraumatology.
